# Genetic diagnosis of familial hypercholesterolaemia by targeted next-generation sequencing

**DOI:** 10.1111/joim.12263

**Published:** 2014-05-21

**Authors:** C Maglio, R M Mancina, B M Motta, M Stef, C Pirazzi, L Palacios, N Askaryar, J Borén, O Wiklund, S Romeo

**Affiliations:** 1 Department of Molecular and Clinical Medicine, Institute of Medicine, Sahlgrenska Academy, Wallenberg Laboratory, University of GothenburgGothenburg, Sweden; 2 Department of Medical and Surgical Sciences, Clinical Nutrition Unit, University Magna Graecia of CatanzaroCatanzaro, Italy; 3 Department of Pathophysiology and Transplantation, University of MilanMilan, Italy; 4 Progenika Biopharma SADerio, Spain

**Keywords:** familial hypercholesterolaemia, genetic diagnosis, LDL receptor, next-generation sequencing, pyrosequencing

## Abstract

Maglio C., Mancina R. M., Motta B. M., Stef M., Pirazzi C., Palacios L., Askaryar N., Borén J., Wiklund O., Romeo S. (University of Gothenburg, Gothenburg, Sweden; University Magna Graecia of Catanzaro, Italy; University of Milan, Italy; Progenika Biopharma SA, Derio, Spain). Genetic diagnosis of familial hypercholesterolaemia by targeted next-generation sequencing.

**Objectives:**

The aim of this study was to combine clinical criteria and next-generation sequencing (pyrosequencing) to establish a diagnosis of familial hypercholesterolaemia (FH).

**Design, setting and subjects:**

A total of 77 subjects with a Dutch Lipid Clinic Network score of ≥3 (possible, probable or definite FH clinical diagnosis) were recruited from the Lipid Clinic at Sahlgrenska Hospital, Gothenburg, Sweden. Next-generation sequencing was performed in all subjects using SEQPRO LIPO RS, a kit that detects mutations in the low-density lipoprotein receptor (*LDLR)*, apolipoprotein B (*APOB*), proprotein convertase subtilisin/kexin type 9 (*PCSK9*) and LDLR adapter protein 1 (*LDLRAP1)* genes; copy-number variations in the *LDLR* gene were also examined.

**Results:**

A total of 26 mutations were detected in 50 subjects (65% success rate). Amongst these, 23 mutations were in the *LDLR* gene, two in the *APOB* gene and one in the *PCSK9* gene. Four mutations with unknown pathogenicity were detected in *LDLR*. Of these, three mutations (Gly505Asp, Ile585Thr and Gln660Arg) have been previously reported in subjects with FH, but their pathogenicity has not been proved. The fourth, a mutation in *LDLR* affecting a splicing site (exon 6–intron 6) has not previously been reported; it was found to segregate with high cholesterol levels in the family of the proband.

**Conclusions:**

Using a combination of clinical criteria and targeted next-generation sequencing, we have achieved FH diagnosis with a high success rate. Furthermore, we identified a new splicing-site mutation in the *LDLR* gene.

## Introduction

Familial hypercholesterolaemia (FH) is a genetic disorder of LDL-C metabolism characterized by elevated levels of LDL-C [1–3]. Individuals with FH are at high risk of premature coronary artery disease, due to lifetime exposure to high levels of circulating LDL-C [4]. Approximately 50% of men and 30% of women with untreated FH are affected by a coronary event by the age of 60 years [2]. The high plasma LDL-C levels induce in some individuals' cholesterol deposition in tendons (i.e. tendon xanthomas) and in the eye (i.e. arcus corneae) [5].

A recent study in a Danish population of 69 016 individuals showed a prevalence of approximately 1/137 for FH diagnosed as definitive or probable according to the Dutch Lipid Clinic Network criteria, suggesting that FH is probably underdiagnosed [4]. The presence of mutations in the LDL receptor (*LDLR)* gene, which is responsible for the cellular uptake of LDL-C, is the most common cause of FH with more than 1000 variants reported [1,6]. Mutations in the apolipoprotein B (*APOB*) [7,8] and proprotein convertase subtilisin/kexin type 9 (*PCSK9*) [9] genes have also been described. *APOB* encodes the principal apolipoprotein of LDL particles that binds to the LDLR [8], whilst PCSK9 protein is involved in LDLR degradation [9]. A recessive form of FH is caused by mutations in the LDLR adapter protein 1 (*LDLRAP1)* gene [10].

Intensive treatment with lipid-lowering medications beginning in early in life prevents cardiovascular events, resulting in life expectancy comparable to that of the overall population [11]. Therefore, early identification of individuals with FH is crucial for effective coronary artery disease prevention. FH diagnosis can be achieved through clinical criteria or by genetic diagnosis [3]. There are several diagnostic algorithms for a clinical diagnosis of FH, and amongst these, the Dutch Lipid Clinic Network score is most commonly used [4]. The Dutch Lipid Clinic Network criteria of FH include high plasma LDL-C levels, family history of hypercholesterolaemia, deposition of cholesterol in extravascular tissues and personal or family history of premature cardiovascular disease.

Several techniques are available for the genetic diagnosis of FH, including assay systems designed to detect specific high-frequency mutations. A combined approach is also available including targeted Sanger sequencing followed by detection of deletions/duplications by multiplex ligation-dependent probe amplification of the *LDLR* gene and finally targeted testing of specific mutations in the *APOB* and *PCSK9* genes. It was recently shown that the sensitivity of the different approaches is very variable [12]. Next-generation sequencing techniques including pyrosequencing allow the fast and reliable investigation of virtually every nucleotide in the whole genome or in specific loci of interest [13,14]. Recently, the use of targeted next-generation sequencing techniques has been validated for FH diagnosis [15,16].

In this study, we combined systematic clinical selection and next-generation sequencing and achieved a high FH genetic diagnosis rate in individuals at high risk. We also identified a new splicing-site mutation in the LDLR.

## Materials and methods

### Study subjects

A total of 77 apparently unrelated adults were included in the study. Subjects attended the Lipid Clinic at the Sahlgrenska University Hospital, Gothenburg, Sweden, during the period 2012–2013. In addition, all subjects had a Dutch Lipid Clinic Network score of ≥3 (3–5, possible FH; 6–7, probable FH; ≥8, definite FH).

### Ethical considerations

All subjects gave their written informed consent to participate in the study. The study was approved by the regional ethics committee of Gothenburg (approval number 145-12).

### Data collection

All subjects were given a questionnaire to complete and underwent a physical examination. The data collected included information about family and personal history, drug therapy and habits. Body mass index and blood pressure were measured, and blood samples were collected by venipuncture after a 12-h fast. Serum lipid levels [including total cholesterol, LDL-C, HDL-C and triglycerides] were determined at the Department of Clinical Chemistry, Sahlgrenska University Hospital, using a Cobas® 8000 modular (c701) analyser with reagents from Cobas® (Roche, F. Hoffmann-La Roche AG Konzern Hauptsitz Grenzacherstrasse 124, CH-4070 Basel Schweiz). LDL-C levels before treatment were available in 55 of 77 (71%) subjects. LDL-C levels for those patients in whom the pretreatment values were not available (*n *=* *22) were quantified based on the estimated effect of the different statins [17]. The diagnosis of cardiovascular disease in the probands was confirmed in patient records. The Dutch Lipid Clinic Network score was calculated as previously described [3].

### Sequencing

DNA was isolated from whole blood as previously described [18]. Then, DNA was sequenced using SEQPRO LIPO RS (Progenika Biopharma, Derio, Spain, http://www.progenika.com/), a next-generation sequencing kit designed to detect mutations in *LDLR*, *APOB*, *PCSK9* and *LDLRAP1*. SEQPRO LIPO RS also analyses copy-number variations in the *LDLR* gene. Exons and exon–intron boundaries of *LDLR* (18 exons), *PCSK9* (12 exons) and *LDLRAP1* (9 exons) were pyrosequenced (454 Life Science, Roche). Moreover, regions in the exons 26 and 29 (between the nucleotides 10 416 and 10 779 for exon 26 and between nucleotides 12 987 and 13 221 for exon 29) of the *APOB* gene were also pyrosequenced. Targeted Sanger sequencing was used to detect mutations in family members of the probands.

### Sequencing: sample preparation and validation

The analysis included five steps: (i) amplification of the regions of interest, (ii) patient identification [using multiplex identifiers (MIDs), i.e. 10 nucleotide sequences that are specific for each patient], (iii) library preparation (purification, quantification, pooling and dilution), (iv) clonal amplification and sequencing by synthesis of the samples and finally (v) data analysis. For further details, see supporting information.

Sample validation for SEQPRO LIPO RS has been performed by Progenika Biopharma. Point mutations have been validated in a total of 299 subjects. Amongst these, 34 samples were totally sequenced for *LDLR*, *APOB*, *PCSK9* and *LDLRAP1* by Sanger sequencing, whilst a further 97 samples were only sequenced for *LDLR* and *APOB* exon 26. Single mutation verification was performed in the remaining 168 samples. SEQPRO LIPO RS showed a sensitivity of 99.6% and a specificity of 100% with an accuracy of 99.6% in detecting point mutations. Copy-number variations have been validated in 545 subjects. SEQPRO LIPO RS v1 showed a sensitivity of 98.6% and a specificity of 95.3% with an accuracy of 95.8% in detecting copy-number variations.

### Prediction of mutation effects and species sequence alignment

*In silico* analyses to predict missense mutation effects were performed using the following bioinformatic tools: Polymorphism Phenotyping version 2 (PolyPhen-2; http://genetics.bwh.harvard.edu/pph2/), Sorting Intolerant From Tolerant (SIFT; http://sift.jcvi.org/www/SIFT_enst_submit.html) and MutationTaster and Consensus Deleteriousness score of missense single nucleotide variations (Condel; http://bg.upf.edu/condel/home). Multiple sequence alignment was performed using Clustal Omega (http://www.ebi.ac.uk/Tools/msa/clustalo/).

### Statistical analysis

Continuous variables are presented as means ± standard deviations and categorical variables as numbers and proportions. General linear model analysis was used to compare continuous variables in subjects with and without detected FH-related mutations. *P* values were adjusted for age, gender and body mass index. Serum triglyceride levels were log-transformed before inclusion in the model. Categorical variables were compared using Fisher's exact test. In the study cohort, the number of mutations detected by SEQPRO LIPO RS was compared with the number of mutations that would have been detected if using LIPOchip v9 (Progenika Biopharma) by Fisher's exact test. Statistical analyses were performed using the IBM Statistical Package for Social Sciences (version 19.0; IBM SPSS Inc., Chicago, IL, USA). Two-sided *P* values <0.05 were considered statistically significant.

## Results

### Study subjects

The study cohort included a total of 77 subjects; the characteristics of the participants are shown in Table1. The mean age of the cohort was 51 ± 14 years, and the mean LDL-C level before treatment was 6.9 ± 1.7 mmol L^−1^. The vast majority of the cohort (69 subjects, 90%) was treated with statins; 40 (52%) individuals were treated with ezetimibe (39 with statins plus ezetimibe and one with ezetimibe alone) and seven subjects did not receive any treatment for hypercholesterolaemia due to statin intolerance or poor adherence. A total of 18 subjects (23%) had premature cardiovascular disease, and 22 (29%) showed tendon xanthomas. All subjects had a Dutch Lipid Clinic Network score of ≥3; the majority (57 subjects, 74%) of the cohort was classified with definite or probable FH as defined by a Dutch Lipid Clinic Network score of ≥6.

**Table 1:** Clinical and biochemical characteristics of the study cohort

	Characteristics	Study cohort
	*n*	77
	Men, *n* (%)	38 (49)
	Age, years	51 ± 14
	Body mass index, kg m^−2^	27 ± 3
	Smoking, *n* (%)	7 (9)
Family history	Premature coronary artery disease, *n* (%)	38 (49)
	Tendon xanthoma, *n* (%)	12 (16)
	Hypercholesterolaemia, *n* (%)	67 (87)
Personal history	Premature cardiovascular disease, *n* (%)	18 (23)
	Tendon xanthoma, *n* (%)	22 (29)
	LDL-C before treatment, mmol L^−1^	7.1 ± 1.7
Current lipid levels	Total cholesterol, mmol L^−1^	5.5 ± 1.8
	LDL-C, mmol L^−1^	3.7 ± 1.7
	HDL-C, mmol L^−1^	1.6 ± 0.4
	Triglycerides, mmol L^−1^	1.2 ± 0.8
Therapy	No therapy, *n* (%)	7 (9)
	Statins, *n* (%)	69 (90)
	Ezetimibe, *n* (%)	40 (52)
Dutch score	≥6 (definite or probable FH)	57 (74)

LDL-C, low-density lipoprotein cholesterol; HDL-C, high-density lipoprotein cholesterol.

### FH-related mutations

Familial hypercholesterolaemia-related mutations were detected in 50 of 77 subjects (65%). Tendon xanthoma and a positive family history for this condition were more common in probands with FH-related mutations compared to those without mutations (*P *=* *0.017 and *P *=* *0.006, respectively, Table S1). Subjects with FH-related mutations also had higher LDL-C levels before treatment (*P *<* *0.001, Table S1); the results were similar after exclusion of subjects for whom LDL-C levels were not available before treatment. Whilst undergoing treatment, no differences in LDL-C levels were observed between individuals with and without FH-related mutations, although combined therapy with a statin and ezetimibe was more common among mutation carriers (*P *=* *0.019, Table S1). In addition, the Dutch Lipid Clinic Network score was higher in subjects with FH-related mutations (*P *=* *0.013, Table S1).

### Spectrum of FH-related mutations

**Figure 1:**
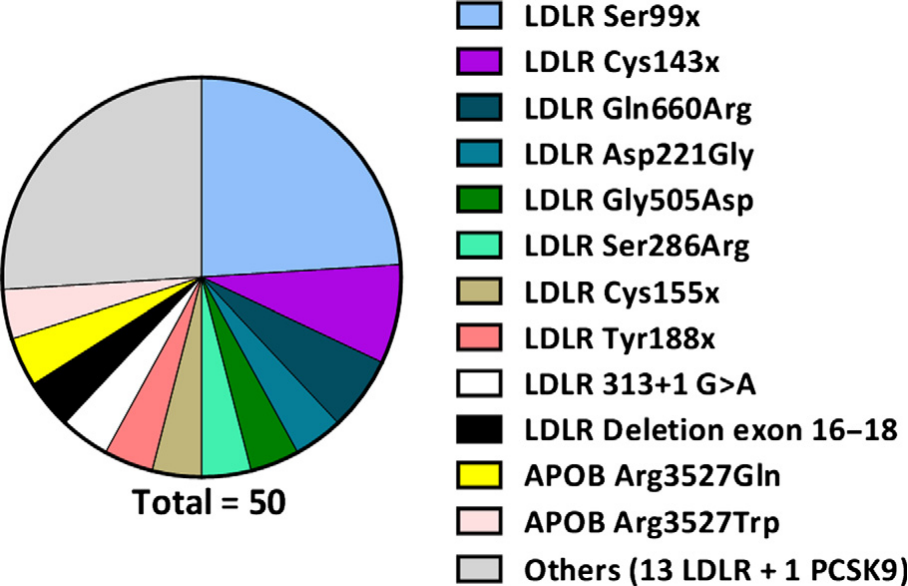
All FH-related mutations detected in the population. Mutations are listed (from top to bottom in descending order) according to their frequency. The most common mutation is *LDLR Ser99x, detected in 12 subjects. The group ‘Others’ includes a total of 14 mutations each detected only in one subject (13 mutations are in LDLR and one in PCSK9*). *LDLR*, *low-density lipoprotein receptor; PCSK9, proprotein convertase subtilisin/kexin type 9; APOB*, *apolipoprotein B; Ser, serine; Cys, cysteine; Gln, glutamine; Arg, arginine; Asp, aspartate; Gly, glycine; Tyr, tyrosine; Trp, tryptophan; x, stop codon*.

Overall, a total of 26 different mutations were detected in 50 subjects (Table S2). Amongst these, 23 mutations (detected in 45 subjects, 90%) were in the *LDLR* gene, two (detected in four subjects, 8%) were in the *APOB* gene and one (detected in one subject, 2%) was in the *PCSK9* gene. No mutations in *LDLRAP1* were found. Amongst the 26 different mutations, almost half (*n *=* *12, 46%) were missense mutations resulting in an amino acid change in the protein sequence, seven (27%) were nonsense (either stop-codon or frameshift variants), three (12%) were splicing-site mutations, and four (15%) were copy-number variations. The most common mutation, found in 12 individuals (24%), was a stop codon at position 99 in the *LDLR* gene (Ser99x, Fig.1). Two different mutations were described in the *APOB* gene: Arg3527Gln (two subjects) and Arg3527Trp (two subjects). One subject had a gain-of-function *PCSK9* mutation (two additional leucines at positions 22 and 23 corresponding to L11 allele: c.61_63 triCTG, p.Leu21 tri, Table S2) [19].

We compared the sensitivity of a customized chip (LIPOchip v9) containing 208 mutations annotated in *LDLR*, three in *APOB* and four in *PCSK9*. Using the LIPOchip, only 50% of the mutations detected by SEQPRO LIPO RS (13/26, *P *<* *0.001) would have been found. Amongst the 13 mutations annotated in the LIPOchip, 11 were in the *LDLR* gene and two in *APOB*. A total of 13 mutations would have been missed using the LIPOchip.

### LDL mutations of unknown pathogenicity

A total of four mutations with uncertain pathogenicity were detected in the *LDLR* gene (Table2). To our knowledge, the splicing mutation (c.940_940+14del15) has not previously been reported, whilst three of these mutations (Gly505Asp, Ile585Thr and Gln660Arg) have been described in subjects screened for high LDL-C levels [20,21], but their pathogenicity has not been formally tested or proved. The glycine to aspartate substitution at position 505 (Gly505Asp, G505D) was present in two different subjects (probands number 1 and 2). These two subjects were later found to be first-degree relatives (Table2). It is interesting that patient number 2, an 82-year-old woman, was homozygous for the Gly505Asp mutation and had pretreatment LDL-C levels of 8.3 mmol L^−1^. A 60-year-old woman (proband number 3) with tendon xanthomas carried a mutation of unknown pathogenicity consisting of an isoleucine to threonine substitution at position 585 (Ile585Thr, I585L) in the *LDLR* gene. We also found an amino acid substitution (glutamine to arginine at position 660, Gln660Arg, Q660R) in the *LDLR* gene in three unrelated subjects. Two of these subjects aged 67 and 59 years (probands number 5 and 6, respectively) had tendon xanthomas, whereas a 27-year-old carrier of the same mutation did not (Table2).

**Table 2:** Characteristics of the study participants with identified mutations with uncertain pathogenicity in the *LDLR* gene

ID	Age, years	Gender	Type	Mutation			LDL-C, mmol L^−1^	Tendon xanthomas	Premature cardiovascular disease
				cDNA	Exon	Amino acid change			
1	59	M	AC	c.1514G>A	10	Gly505Asp	5.7	No	No
2^a^	82	F	AC	c.1514G>A	10	Gly505Asp	8.3	Yes	No
3	60	F	AC	c.1754T>C	12	Ile585Thr	8.6	Yes	No
4	27	M	AC	c1979A>G	13	Gln660Arg	5.6	No	No
5	67	F	AC	c1979A>G	13	Gln660Arg	5.3	Yes	No
6	59	F	AC	c1979A>G	13	Gln660Arg	7.9	Yes	No
7	21	M	Splicing	c.940_940+14del15	6	–	7.0	No	Yes

a Homozygous and related (consanguinity) to subject number 1; heterozygous for the same mutation.

LDL-C levels refer to measurements before starting lipid-lowering medications.

LDLR, low-density lipoprotein receptor; ID, identification number; F, female; M, male; AC, amino acid change; Gly, glycine; Asp, aspartate; Ile, isoleucine; Thr, threonine; Gln, glutamine; Arg, arginine.

The alignment of the amino acids in the LDLR across eight different animal species, ranging from *Homo sapiens* to *Danio rerio* (zebra fish), showed that three amino acids (in humans: Gly505, Ile585 and Gln660) are highly preserved in the different species (Fig. S1). Moreover, the *in silico* analysis performed with four different prediction tools suggested that all the three variants are pathogenic (Table S3).

### *Novel* LDLR *mutation*

A previously unreported mutation of unknown pathogenicity was detected in the *LDLR* gene in one subject (proband number 7). This subject carried a mutation (c.940_940+14del15) caused by a 15-nucleotide deletion from the last nucleotide of exon 6 (position 940 of the cDNA) to the 14th nucleotide of intron 6. This microdeletion is likely to affect protein splicing and impair protein function. The subject was a 21-year-old man who developed acute myocardial infarction at the age of 16 years. He had a family history of cardiovascular disease and hypercholesterolaemia and was a smoker. Before treatment, his serum LDL-C level was 7.0 mmol L^−1^ and body mass index was 35.8 kg m^−2^. At the age of 18 years, following the myocardial infarction, he underwent bariatric surgery for weight loss and at the time of the last observation had a body mass index of 23.9 kg m^−2^. The current therapy for this subject includes rosuvastatin (40 mg) and ezetimibe; his last reported serum LDL-C level was 1.5 mmol L^−1^.

**Figure 2:**
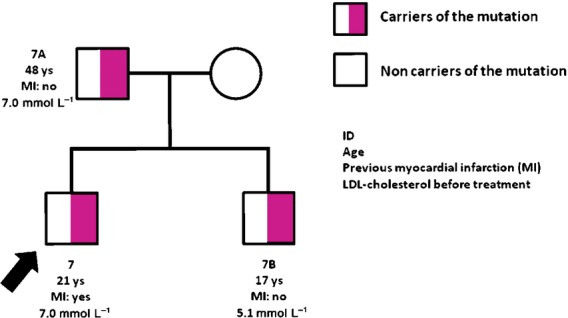
Pedigree of proband number 7 (c.940_940+14del15 splicing mutation). LDL-C levels refer to measurements before starting lipid-lowering medications. The arrow indicates the index case. Half-shaded squares indicate male carriers of the mutation; open circles indicate female noncarriers of the mutation. ID, identification number; ys, years.

DNA from the first-degree relatives of proband number 7 was extracted and sequenced to look for the splicing mutation (c.940_940+14del15) found in the proband (Fig.2). The splicing mutation was detected in both his father and his brother. His father (7A) was a 48-year-old man without cardiovascular disease followed at the Lipid Clinic at the Sahlgrenska Hospital for hypercholesterolaemia (serum LDL-C level before treatment was 7.0 mmol L^−1^). His brother (7B) was 17 years old and had hypercholesterolaemia (LDL-C of 5.1 mmol L^−1^) but no previous cardiovascular disease. The splicing mutation was not detected in the proband's mother whose LDL-C levels were within the normal range.

## Discussion

Genetic diagnosis is the gold standard to identify individuals with FH. Genetic diagnosis is clinically relevant especially for family screening in individuals with borderline/moderately high serum LDL-C levels. In the present study, we obtained a high FH diagnosis success rate by combining targeted next-generation sequencing and clinical criteria. Furthermore, we identified a new causative mutation: a splicing-site variant in the *LDLR* gene.

We screened a total of 77 individuals with a Dutch Lipid Clinic Network score of ≥3, corresponding to possible, probable or definite FH diagnosis [3], and targeted pyrosequencing of the exons and exon–intron boundaries was performed in the *LDLR, PCSK9* and *LDLRAP1* genes and in *APOB* exons 26 and 29, including the LDLR-binding domain. Amongst these 77 individuals, 50 (65%) carriers of FH-related mutations in *LDLR, APOB* or *PCSK9* were identified.

Several diagnostic tools are now available for FH diagnosis, ranging from simple chip assay systems to complex combined approaches [12]. The sensitivity of the different techniques is extremely variable depending on the method used and on the inclusion criteria of the patients. The results of a recent systematic review by Sharma *et al*. suggested that a comprehensive genetic analysis performed by direct sequencing of the *LDLR* gene and multiplex ligation-dependent probe amplification followed by detection of specific frequent mutations in *APOB* and *PCSK9* is an accurate approach for FH genetic diagnosis [12]. On the other hand, because this approach is time-consuming and expensive, the authors postulated that next-generation sequencing techniques will enable more efficient and cost-effective diagnosis of FH [12].

In the present study, pyrosequencing was used effectively as a first-line screening test to detect FH-related mutations. Amongst the 77 individuals fulfilling the clinical criteria for FH, 50 (65%) were found to have a mutation in one of the following genes: *LDLR*, *APOB* or *PCSK9*. None of the subjects had a mutation in *LDLRAP1*. As expected, xanthomas were more common and pretreatment LDL-C levels were higher in individuals with FH-related mutations compared to those without these mutations. Serum LDL-C levels were not different between groups during treatment, but combination therapy with a statin and ezetimibe was more common amongst subjects with FH-related mutations, indicating that more intensive treatment is required to control lipid levels in these individuals.

Recently, next-generation sequencing has been successfully used to detect mutations in the entire *APOB* gene in subjects with a clinical diagnosis of FH and no evidence of mutations in *LDLR* or *PCSK9,* or in *APOB* exons 26 and 29 [22]. Moreover, next-generation sequencing was used in two previous studies for FH diagnosis in cohorts selected for high cholesterol levels, and diagnosis success rates of 36% [23] and 26% [24] were reported. In the present study, we have systematically selected probands using a Dutch Lipid Clinic Network score of ≥3, thus obtaining approximately a twofold increase in the genetic diagnosis rate (up to 65%). This suggests that the combination of systematic selection of probands and next-generation sequencing technology results in a high diagnosis success rate. This is particularly important if next-generation techniques are to be used for FH diagnosis in clinical settings and healthcare systems.

We also compared the mutations we found with SEQPRO LIPO RS to those that would have been detected if using LIPOchip. Only half of the mutations present in our population would have been found by LIPOchip. It is important to note that samples in which a mutation has not been detected by LIPOchip would have needed a full *LDLR* gene resequencing for diagnosis. This suggests that precustomized assays for FH mutations are of limited use unless the spectrum of FH-related mutations present in a specific geographical region is already well known.

Several variants detected in our cohort were well-known mutations previously described in Scandinavian populations [20,25]. We found a total of four mutations with uncertain pathogenicity. Amongst these, three have been described previously in European cohorts with hypercholesterolaemia [20,21]. The *in silico* analyses and the high degree of conservation across species suggest that the mutations are loss of function. Taken together, these data suggest that the three FH mutations are indeed causative.

Of interest, proband number 2 was homozygous for the Gly505Asp mutation in *LDLR* but had a milder phenotype than expected. The same mutation has been previously described in three individuals living in the Gothenburg area [20]. Two of these subjects had a homozygous mutation but did not present a typical homozygous phenotype. We hypothesize that this mutation does not fully abolish the activity of the LDLR resulting in some residual function of the receptor.

We also identified a new mutation in the exon 6 splicing site (940_940+14del15). This mutation is likely to severely affect mRNA splicing and protein function. Furthermore, this FH-related mutation segregates with high serum LDL-C levels within the family, suggesting that this is a causative mutation. The proband phenotype was severe with a premature myocardial infarction at the age of 16 years. However, it should be noted that the patient had multiple risk factors for myocardial infarction including smoking and degree II obesity.

Next-generation sequencing of the entire genome has already been used in family-based genetic studies [14,26]. However, resequencing of the entire genome is expensive, laborious and time-consuming, especially when this approach is extended to large cohorts. Moreover, it may raise some ethical issues regarding the investigation of mutations responsible for diseases for which there is no preventive treatment. This is not the case with targeted next-generation sequencing in which only genes specifically involved in FH pathogenesis are screened. Furthermore, the targeted sequencing of FH-related genes is an affordable and rapid technique.

A limitation of this study is that untreated LDL-C levels were not retrieved for 29% of the individuals in whom pretreatment LDL-C levels were estimated [17]. Another limitation is that family history was based on patient interview, which is self-reported and dependent on contact between patients and their family members.

In summary, we obtained a high success rate of FH diagnosis by combining next-generation sequencing and clinical criteria in individuals with hypercholesterolaemia. We have also identified a previously unknown splicing-site mutation in the *LDLR* gene.

## Conflict of interest statement

MS and LP are employed full-time by Progenika Biopharma SA. All other authors have no conflicts of interest to declare.

## Supporting Information

**Data S1.**Methods. **Table S1.** Clinical and biochemical characteristics of the study cohort stratified by the FH-related mutations. **Table S2.** Mutations identified in the *LDLR*, *APOB* or *PCSK9* genes. **Table S3.** *In silico* prediction of effect for the nonsynonymous mutations with unknown pathogenicity.
