# Supplementary material for: Genetic diagnosis of familial hypercholesterolaemia by targeted next-generation sequencing
Source: J Intern Med. 2014 May 21;276(4):396–403. doi: 10.1111/joim.12263 (PMC4369133; doi:10.1111/joim.12263)
Supplement: Supplementary file 1 — Data S1.Methods. Table S1. Clinical and biochemical characteristics of the study cohort stratified by the FH-related mutations. Table S2. Mutations identified in the LDLR, APOB or PCSK9 genes. Table S3. In silico prediction of effect for the nonsynonymous mutations with unknown pathogenicity. [file joim0276-0396-sd1.doc]

**SUPPORTING INFORMATION**

**Supporting methods**

*Sequencing- samples preparation*

The first step in the samples preparation is the amplification of the regions of interest. The Universal tailed amplicon sequencing strategy from Roche (<http://454.com/downloads/my454/applications-info/454SequencingSystemGuidelinesforAmpliconExperimentalDesign_March2012.pdf>) was followed for the preparation of the sample library. Two consecutive amplifications, the first one to amplify the target regions and the second to include the index or MID allowing the identification of the samples were performed in order to amplify target regions in various samples and to prepare them for the clonal amplification and the sequencing processes. In brief, 44 amplicons covering the regions of interest as well as 4 amplicons covering chromosome 21 regions involved in Down syndrome phenotype were grouped in 4 multiplex PCRs and were amplified under the same condition for the first and the second PCR. Between the 2 PCRs a dilution was performed and after the amplifications steps, the 4 PCR for each sample were pooled using an Ampure XP beads (Agencourt) ratio of 2:1 (PCR/Beads). After purification and dilution at 10^6^ molecules/µL, amplicons were mixed with the sequencing beads at a ratio of 1:1. The clonal amplification or emulsion PCR as well as the sequencing process were carried out according to the manufacturer instructions (<http://454.com/downloads/my454/documentation/gs-junior/method-manuals/GSJunioremPCRAmplificationMethodManualLib-A_March2012.pdf> and <http://454.com/downloads/my454/documentation/gs-junior/method-manuals/GSJuniorSequencingManual_Jan2013.pdf> respectively). To sequence the samples, each amplicon undergoes a clonal amplification on beads, which will then be subjected to pyrosequencing. Light emitted by each new nucleotide incorporation is collected by the GS Junior and converted in sequences by the Roche Amplicon Variant Analyzer software (AVA). Data analysis was performed with the SEQPRO LIPO RS software that automatically launched the Roche 454 AVA software for alignment and variant detection. Coverage of at least 10 forward and 10 reverse reads was required for high reliability variant calling. Variant were named according to the HGVS nomenclature and compared to Progenika Database to classify the variants as true variants, potential false positives or false positives. New variants that were not present in the Progenika’s database and potential false positives are confirmed by Capillary Electrophoresis sequencing. Results were analyzed by two trained technologists and a supervisor. For CNV detection, based on coverage depth, proprietary algorithms were applied.

**Supplemental** **Table 1**. Clinical and biochemical characteristics of the study cohort stratified by the FH-related mutations.

|  | **Characteristics** | **Mutation** | **No mutation** | **P value** |
| --- | --- | --- | --- | --- |
|  | **N** | 50 | 27 | - |
|  | **Men, n (%)** | 24 (48%) | 14 (52%) | 0.814 |
|  | **Age, years** | 49±14 | 54±13 | 0.177 |
|  | **Body-mass index, Kg/m^2^** | 27±4 | 27±3 | 0.792 |
|  | **Smoking, n (%)** | 6 (12%) | 1 (4%) | 0.411 |
| **Family history:** | **Premature coronary artery disease, n (%)** | 28 (56%) | 10 (37%) | 0.153 |
|  | **Tendon xanthomas, n (%)** | 12 (24%) | 0 (0%) | 0.006 |
|  | **Hypercholesterolemia, n (%)** | 44 (88%) | 23 (85%) | 0.734 |
| **Personal history:** | **Premature cardiovascular disease, n (%)** | 12 (24%) | 6 (22%) | 0.999 |
|  | **Tendon xanthomas, n (%)** | 19 (38%) | 3 (11%) | 0.017 |
|  | **LDL-C before treatment, mmol/L** | 7.7±1.7 | 6.1±1.1 | <0.001* |
| **Current lipids:** | **Total Cholesterol, mmol/L** | 5.5±1.9 | 5.5±1.8 | 0.763 |
|  | **LDL-C, mmol/L** | 3.7±1.7 | 3.6±1.6 | 0.696 |
|  | **HDL-C, mmol/L** | 1.6±0.4 | 1.7±0.4 | 0.687 |
|  | **Triglycerides, mmol/L** | 1.0±0.4 | 1.6±1.1 | 0.003 |
| **Therapy:** | **No therapy, n (%)** | 1 (2%) | 6 (22%) | 0.007 |
|  | **Statins, n (%)** | 49 (98%) | 20 (74%) | 0.002 |
|  | **Ezetimibe, n (%)** | 31 (62%) | 9 (33%) | 0.019 |
| **Dutch score:** | **≥6 (definite or probable FH)** | 42 (84%) | 15 (56%) | 0.013 |

P values have been calculated by Fisher exact test (categorical variables) or general linear model analysis adjusted for age, gender and body-mass index, when appropriate (continuous variables). *virtually unchanged when subjects without available LDL-C levels before treatment are excluded. Serum triglycerides have been log-transformed before entering the model.

*Abbreviations*: LDL-C, low-density lipoprotein cholesterol; HDL-C, high-density lipoprotein cholesterol.

**Supplemental** **Table 2.** Mutations identified in the *LDLR, APOB* or *PCSK9* genes.

| **Gene** | **Mutation type** | **Mutation** | **Exon/intron** | **Pathogenicity** | **# subjects** |
| --- | --- | --- | --- | --- | --- |
| LDLR | Aminoacid change | Trp87Gly | Exon 3 | Known | 1 |
|  |  | Asp221Gly | Exon 4 | Known | 2 |
|  |  | Ser286Arg | Exon 6 | Known | 2 |
|  |  | Glu357Lys | Exon 8 | Known | 1 |
|  |  | Glu408Lys | Exon 9 | Known | 1 |
|  |  | Gly505Asp | Exon 10 | Unknown | 2* |
|  |  | Gly549Asp | Exon 11 | Known | 1 |
|  |  | Ile585Thr | Exon 12 | Unknown | 1 |
|  |  | Gln660Arg | Exon 13 | Unknown | 3 |
|  |  | Pro685Leu | Exon 14 | Known | 1 |
|  | Stop codon | Ser99x | Exon 3 | Known | 12 |
|  |  | Cys143x | Exon 4 | Known | 4 |
|  |  | Cys155x | Exon 4 | Known | 2 |
|  |  | Tyr188x | Exon 4 | Known | 2 |
|  |  | Cys296x | Exon 6 | Known | 1 |
|  |  | Trp813x | Exon17 | Known | 1 |
|  | Deletion | Promoter and Exon 1 | Exon 1 | Known | 1 |
|  |  | Gly219 del | Exon 4 | Known | 1 |
|  |  | Exon 16 to 18 | Exon 16-18 | Known | 2 |
|  | Frameshift | His690ThrFS | Exon 14 | Known | 1 |
|  | Splicing | 313+1 G>A | Intron 3 | Known | 2 |
|  |  | 940_940+14del15 | Exon 6 | Unknown | 1 |
|  |  | 2390-2A>G | Intron 16 | Known | 1 |
| APOB | Aminoacid change | Arg3527Gln | Exon 26 | Known | 2 |
|  |  | Arg3527Trp | Exon 26 | Known | 2 |
| PCSK9 | **Triplication** | **Leu 21 tri** | Exon 1 | Known | 1 |

* Two consanguineous subjects. One is homozygous for the reported mutation.

*Abbreviations*: LDLR, low-density lipoprotein receptor; APOB, apolipoprotein B; PCSK9, proprotein convertase subtilisin/kexin type 9; Asp, aspartate; Gly, glycine; Gln, glutamine; Arg, arginine; Glu, glutamate; Lys, lysine; Ile, isoleucine; Thr, threonine; Pro, proline; Leu, leucine; Ser, serine; Cys, cysteine; Tyr, tyrosine; Trp, tryptophan; His, histidine.

**Supplemental Table 3. *In silico* prediction of effect for the nonsynonymous mutations with unknown pathogenicity**

| **Mutation** | | | **PolyPhen-2** | **SIFT** | **Mutation Taster** | **Condel** |
| --- | --- | --- | --- | --- | --- | --- |
| **cDNA** | **Exon** | **Aminoacidic change** |
| c.1514G>A | 10 | Gly505Asp | Probably damaging | Damaging | Disease causing | Deleterious |
| c.1754T>C | 12 | Ile585Thr | Possibly damaging | Damaging | Disease causing | Deleterious |
| c1979A>G | 13 | Gln660Arg | Probably damaging | Damaging | Disease causing | Deleterious |

*Abbreviations*: Ile, isoleucine; Thr, threonine; Gly, glycine; Asp, aspartate Gln, glutamine; Arg, arginine; PolyPhen-2, Polymorphism Phenotyping version 2; SIFT, Sorting intolerant from tolerant; Condel, Consensus deleteriousness score of missense single-nucleotide variations.

**
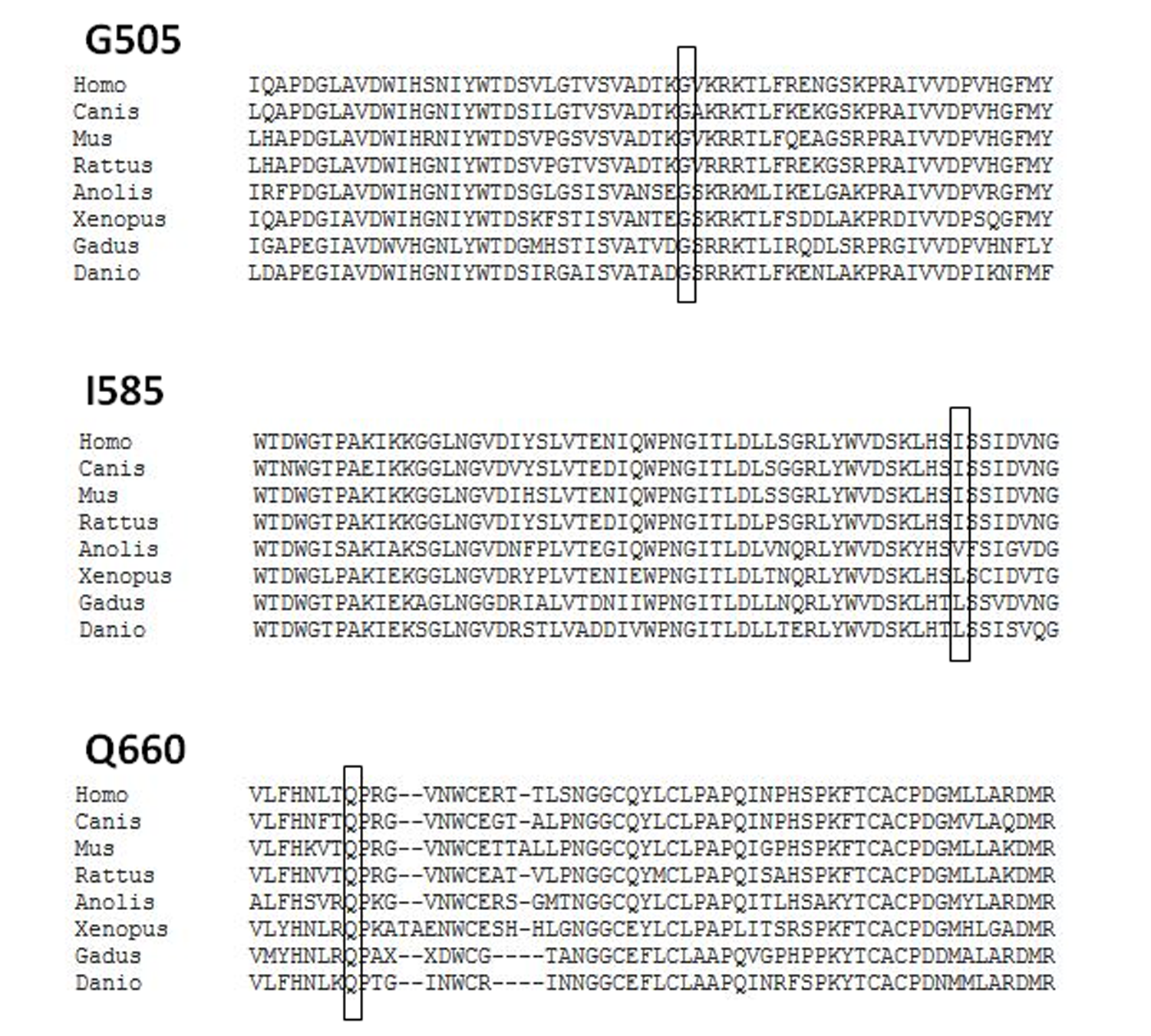
**

**Supplementary Figure 1**. Alignments of the LDLR protein in 8 different species.

The alignment of the aminoacids of the LDLR protein in 8 different species is shown. The species include: Homo sapiens, Canis lupus familiaris, Mus musculus, Rattus norvegicus, Anolis carolinensis, Xenopus tropicalis, Gadus morhua, Danio rerio.

*Abbreviations*: G, glycine; I, isoleucine; Q, glutamine.
